# Syotti: scalable bait design for DNA enrichment

**DOI:** 10.1093/bioinformatics/btac226

**Published:** 2022-06-27

**Authors:** Jarno N Alanko, Ilya B Slizovskiy, Daniel Lokshtanov, Travis Gagie, Noelle R Noyes, Christina Boucher

**Affiliations:** Department of Computer Science, University of Helsinki, Helsinki, Finland; Faculty of Computer Science, Dalhousie University, Halifax, Canada; Department of Veterinary Population Medicine, University of Minnesota, St. Paul, MN, USA; Department of Computer Science, University of California, Santa Barbara, CA, USA; Faculty of Computer Science, Dalhousie University, Halifax, Canada; Department of Veterinary Population Medicine, University of Minnesota, St. Paul, MN, USA; Department of Computer and Information Science and Engineering, University of Florida, Gainesville, FL, USA

## Abstract

**Motivation:**

Bait enrichment is a protocol that is becoming increasingly ubiquitous as it has been shown to successfully amplify regions of interest in metagenomic samples. In this method, a set of synthetic probes (‘baits’) are designed, manufactured and applied to fragmented metagenomic DNA. The probes bind to the fragmented DNA and any unbound DNA is rinsed away, leaving the bound fragments to be amplified for sequencing. Metsky *et al.* demonstrated that bait-enrichment is capable of detecting a large number of human viral pathogens within metagenomic samples.

**Results:**

We formalize the problem of designing baits by defining the Minimum Bait Cover problem, show that the problem is NP-hard even under very restrictive assumptions, and design an efficient heuristic that takes advantage of succinct data structures. We refer to our method as Syotti. The running time of Syotti shows linear scaling in practice, running at least an order of magnitude faster than state-of-the-art methods, including the method of Metsky *et al.* At the same time, our method produces bait sets that are smaller than the ones produced by the competing methods, while also leaving fewer positions uncovered. Lastly, we show that Syotti requires only 25 min to design baits for a dataset comprised of 3 billion nucleotides from 1000 related bacterial substrains, whereas the method of Metsky *et al.* shows clearly super-linear running time and fails to process even a subset of 17% of the data in 72 h.

**Availability and implementation:**

https://github.com/jnalanko/syotti.

**Supplementary information:**

Supplementary data are available at *Bioinformatics* online.

## 1 Introduction

Our understanding of microbial species has evolved at an impressive rate, but it is estimated that 99% of micro-oganisms cannot live outside their natural environments, and thus, cannot be cultured and sequenced (Palkova, 2004). This created a significant roadblock in studying such species. In the mid-2000s, metagenomic shotgun sequencing became widely available, which enabled the sequencing of all of the DNA within non-cultured samples—whether a swab of a person’s mouth or a soil sample. This enabled the large-scale study of micro-organisms, and thus greatly expanded our knowledge in this field. However, many scientific questions focus on specific sequences such as antimicrobial resistance (AMR) genes (Noyes *et al.*, 2017), or human viral strains and substrains (Deng *et al.*, 2020). To address these questions, most of the sequence reads in a metagenomic dataset are irrelevant, and are typically eliminated from further consideration. Moreover, the percentage of such reads can be quite high; for instance in agricultural samples, 90–95% of reads are often eliminated because they are not of interest to the research question (Guitor *et al.*, 2019; Noyes *et al.*, 2017; Rubiola *et al.*, 2020). Furthermore, using shotgun metagenomics in these scenarios not only leads to undue monetary expense but also greatly lowers the sensitivity of detection. For example, Lee *et al.* (2017) demonstrated that out of 31 viral strains identified, 11 were unidentified via traditional sequencing, and Noyes *et al.* (2017) reported that 1441 AMR genes within agricultural samples were undetected by standard metagenomic sequencing.

One way to address these issues is to move beyond sequencing the entire microbial population within a sample, and rather to select the sequences of interest for targeted sequencing. This can be accomplished through *targeted enrichment*, which uses *biotinylated cDNA bait molecules* to target-specific regions from DNA libraries for sequencing. More specifically, baits (short synthetically created single-stranded cDNA molecules) bind to their DNA targets and are then captured within the sample using a magnet. Non-captured, unbound DNA fragments (i.e. non-targeted DNA) are then rinsed away. Thus, only bound—i.e. targeted—DNA is sequenced. Although non-targeted DNA is not completely eliminated from sequencing, it is greatly reduced. One of the first, and arguably the most critical steps within this process requires solving a computational problem: for a given set of target DNA sequences (e.g. set of genes or viral strains) and a specified bait length *k*, identify a set of baits such that there exists at least one bait in that set that binds to each position of every sequence in the database.

Initially one could expect that baits could be computationally designed by finding all unique *k*-length subsequences (*k*-mers) in the targeted DNA. This solution however is not feasible as the number of *k*-mers grows rapidly with increasing dataset size. Thus, two key challenges need to be addressed for effective bait design. First, the number of baits must be minimized, as the cost of targeted enrichment is proportional to the size of the bait set and there is an upper limit set by the bait manufacturer. For example, Agilent Technologies Inc. sets a limit of ∼220 000 baits, implying that larger bait sets can be manufactured but would need to be split into multiple sets—thus, increasing the cost and labor of purchasing and processing multiple bait sets per sample. Second, the baits do not bind exactly to a single DNA sequence identical to that bait, but will bind to any subsequence with some allowed number of mismatches between the bait and target DNA sequence (typically, over 70% of positions must match). For this reason, computationally designing effective bait sets is a challenging problem, which to the best of our knowledge, has not been formally defined or considered from an algorithmic perspective. Although there are a number of methods for designing baits—including MrBait (Chafin *et al.*, 2018), CATCH (Metsky *et al.*, 2019) and BaitFisher (Mayer *et al.*, 2016)—most methods are unable to scale or provide reasonable output to even moderate-sized sets of genomes.

We formalize this problem, which we call The Minimum Bait Cover Problem, and show it is NP-hard even for extremely restrictive versions of the problem. For example, the problem remains intractable even for an alphabet of size four (i.e. A, T, C, G), a single reference genome, a bait length that is logarithmic in the length of the reference genome, and Hamming distance of zero. In light of these hardness results, we provide an efficient heuristic based on the FM-index and demonstrate that it is capable of scaling to large sets of sequences. We refer to our method as Syotti (Finnish word for ‘Bait’, with the letter ö replaced with the letter o) and compare it to all competing methods on three datasets: (i) MEGARes, which is a database of AMR genes, (ii) a set of genomes corresponding to substrains of *Salmonella enterica* subsp. enterica, *Escherichia coli*, *Enterococcus* spp. and *Campylobacter jejuni* and (iii) a set of viral genomes. We determined that Syotti is the only method capable of scaling to the second and third datasets. MrBait was able to process all datasets, but was over 20 times slower and produced over 40 times more baits than Syotti, making it unreasonable in practice. CATCH failed to process both the second and third dataset in 72 h, and even failed to process prefixes of length 17% and 5% respectively in the same time limit. The curve of the running time of CATCH indicates that it would take at least 20 days to process the viral dataset, and probably much longer.

Lastly, we evaluated the coverage of the genomes with respect to the bait sets using our implementation of the evaluation method provided with CATCH (Metsky *et al.*, 2019). On the smallest dataset (MEGARes), both Syotti and CATCH covered 100.0% of the nucleotide positions at least once, but MrBait covered only 96.4%. On the largest subset of bacteria genomes that all tools were able to run, Syotti produced 158 thousand baits, CATCH produced 241 thousand and MrBait over 1 million; hence, the bait set of MrBait was too large to be deemed of any practical use. Lastly, we considered the full set of viral genomes and compared the baits of Syotti against the published and publicly available bait sets of size 250k, 350k and 700k published with CATCH. The coverage of the CATCH bait sets were 84.1%, 90.6% and 97.5%, respectively. The coverage of Syotti was 99.5%, with the remaining 0.5% being due to unknown N-characters in the data.

## 2 Related work

Given this is relatively a new computational problem, there are few methods for bait design, which we now summarize. CATCH was released by Metsky *et al.* (2019). The algorithm first generates a set of candidate baits by sliding a window of length *L* with a stride of *s* over the input sequences. Duplicate and near-duplicate candidates may be filtered out by using a locality sensitive hash function (LSH). The software offers two different options for the LSH: one based on Hamming distance, and other using the Minimizer hashing. Next, the candidate baits are mapped to the target sequences using a seed-and-extend approach.

A tunable hybridization model is used to determine whether a bait matches to the mapped position. The user can tune the model with the following three ways: (i) allow a given number of mismatches to be tolerated; (ii) require that there be an exact match of a given length; and (iii) define that a bait hybridizes to the target if at least one of its substrings of a given length hybridizes to the target. After all sequences are mapped and the hybridization verified according to the specified model, CATCH filters redundant candidate baits. This is done by reducing the problem into an instance of the classical Set Cover problem. The input to the Set Cover instance is a family of subsets of positions in the input sequences. The Set Cover instance is solved using a greedy heuristic. If the bait design algorithm of CATCH is applied across diverse taxa, it may result in over-representation of the more diverse taxa in the bait set. To address this issue, the user can specify a different weight for each taxon. CATCH then runs a constrained non-linear optimization algorithm using a truncated Newton algorithm, enforcing the constraints with the *barrier method*. The aim is to optimize different hybridization parameters for each taxon that minimize a weighted loss-function, penalizing less stringent hybridization models.

BaitFisher (Mayer *et al.*, 2016) is another method for bait design. It takes as input a multiple alignment of the target sequences, and then clusters the input sequences according to Hamming distance, and constructs a consensus sequence for each cluster. The consensus sequence is constructed to minimize the maximum Hamming distance to all sequences in the cluster. It is computed with either an exhaustive brute force method, or approximated via a greedy heuristic. BaitFisher constructs a provisional bait set by tiling the consensus sequences with baits. The bait set is post-processed by a helper program called BaitFilter to remove possible issues such as baits binding to multiple regions.

MrBait (Chafin *et al.*, 2018) designs baits by first detecting and filtering suitable target regions depending on use specification, and then tiling target regions with baits. As a postprocessing step, baits may be filtered to remove redundancy, by computing pairwise alignments of the baits and finding a maximal independent set of baits.

Other tools for bait design include AnthOligo (Jayaraman *et al.*, 2020), MetCap (Kushwaha *et al.*, 2015) and BaitsTools (Campana, 2018). We do not consider these tools because former two are only offered through a web-interface, and BaitsTools uses a similar tiling method to MrBait.

##  

### 3 The minimum bait cover problem

#### 3.1 Preliminaries

We define a string *S* as a finite sequence of characters S=S[1 …n] over an alphabet $\Sigma =\{c_{1},\;\ldots ,c_{\sigma }\}$. We denote the length of a string *S* by |S|, and the empty string by *ε*. We denote by S[1 …i] the *i*-th prefix of *S*, and by S[i …n] the *i*-th suffix of *S*. Given two strings *S* and *T*, we say that *S* is lexicographically smaller than *T* if either *S* is a prefix of *T* or there exists an index i≥1 such that S[1 …i]=T[1 …i] and S[i+1] occurs before T[i+1] in Σ. We denote this as S≺T. Next, we define the *Hamming distance* between *S* and *T* (which have the same length) as the number of positions where *S* and *T* mismatch, namely d(S,T):=|{i:S[i]≠T[i]}|. Lastly, we denote S°T for the string concatenation of *S* and *T*. Given a set of strings, $S=\{S_{1},..S_{m}\}$, we denote the total length $\sum_{S\in S}|S|$ of all strings in S by ‖S‖.

For a string *S* and integer ℓ≤|S| we denote by pre(S,ℓ) the prefix S[1,…,ℓ] of *S* of length ℓ. Similarly, we denote by suf(S,ℓ) the suffix S[ℓ−ℓ+1,…,ℓ] of *S* of length ℓ.

#### 3.2 Hardness results

We fix the length of the bait sequences to a constant *L*. We say a string *X covers* a position i≤|Y| in a string *Y* if there exists a *j* such that i−|X|<j≤i and X=Y[j,j+1,…j+|X|−1]. More generally, *X distance θ-covers* (we will just say *θ*-*covers*) position *i* in *Y* if there exists a *j* such that i−|X|<j≤i and d(X,Y[j,j+1,…j+|X|−1])≤θ. Observe that *X* covers *i* if and only if *X* 0-covers *i*. A set T of strings *θ*-cover a string *S* if, for every position *i* in *S* there exists a string T∈T that *θ*-covers *i*. A set T of strings *θ*-cover a set S of strings if every string S∈S is *θ*-covered by T. We are now ready to define the main problem considered in this article, namely Minimum Bait Cover.

Minimum Bait Cover

Input: Here the input consists of two integer parameters θ≥0 and *L *>* *0 and a set S of *n* strings $S=\{S_{1},\ldots ,S_{n}\}$ over a finite alphabet Σ.

Question: What is the smallest possible set T of strings, each of length exactly *L*, such that S is *θ*-covered by T. 

In the closely related String Cover problem input is a single string *S* and a parameter *L *>* *0. The task is to find a minimum size set T of strings, each of length exactly *L*, such that *S* is *θ*-covered by T. Comparing the two problem definitions it is easy to see that String Cover is precisely equal to the special case of Minimum Bait Cover when *n *=* *1 and *θ* = 0. Cole *et al.* (2005) proved that String Cover is NP-hard even for *L *=* *2. This immediately leads for the following hardness result for Minimum Bait Cover.

Proposition 3.1 (Cole *et al.*, 2005) *(a) String Cover is NP-hard even for L = 2. (b) Minimum Bait Cover is NP-hard, even for n = 1, θ = 0 and L = 2.*

Proposition 3.1 effectively rules out any hope of a polynomial time algorithm, or an efficient (Fixed Parameter Tractable or Slicewise Polynomial) parameterized algorithm with parameters *n*, *θ* and *L*. At the same time the instances of String Cover constructed in the reduction of Cole *et al.* (2005) are strings over a large alphabet |Σ|. Since we are primarily interested in instances of Minimum Bait Cover with |Σ|=4, this might leave some hope that for small alphabets one can still obtain efficient parameterized algorithms. We now show that (almost) all of the hardness from Proposition 3.1 is retained even when |Σ| is constant.

Theorem 3.2 (a) *For every* k≥2, *String Cover is NP-hard with* |Σ|=k  *and* L=O(log |S|). (b) *For every* k≥2, Minimum Bait Cover *is NP-hard, even for* |Σ|=k, S={S}, *θ *= 0 *and* L=O(log |S|).

Theorem 3.2 shows that unless P=NP there cannot exist an algorithm for Minimum Bait Cover with running time $2^{O(L)}\|S{\|}^{g(|\Sigma |,n,\theta )}$ for any function *g* of constant parameters |Σ|,n and *θ*. We refer the reader to Chapter 1 of Downey and Fellows (2012) for a more thorough discussion fixed parameterized complexity, W[1]-hardness and NP-hardness. This effectively rules out parameterized algorithms that exploit any of the most natural parameters one could expect to be small in relevant input instances. We remark that part (a) of Theorem 3.2 resolves in the affirmative a conjecture of Cole *et al.* (2005) that String Cover remains NP-hard even with a constant size alphabet.

Proof of Theorem 3.2 We only prove (*a*), since (*b*) follows from (*a*) together with the fact that String Cover is the special case of Minimum Bait Cover with *θ* = 0 and *n *=* *1. Fix an integer k≥2. We give a reduction from String Cover with *L *=* *2 and unbounded alphabet size (i.e. |Σ|≤|S|) to String Cover with alphabet $\Sigma_{k}=\{0,\ldots ,k-1\}$ of size exactly *k*.

We now describe the construction. Given as input an instance (Σ,S,L=2) of String Cover the reduction algorithm sets $\Sigma_{k}=\{0,\ldots ,k-1\},\;\ell$ to be the smallest integer above $\left\lfloor 100{\;\text{log}\;}_{2}(|\Sigma |)\right\rfloor$ that is divisible by 3, and L′=2ℓ. The algorithm then computes a set of strings $\left\{S_{a}\;:\;a\in \Sigma \right\}$ by using Claim 3.3.

Claim 3.3 There exists an algorithm that given as input a set Σ, runs in time polynomial in |Σ|, and outputs a set $\left\{S_{a}\;:\;a\in \Sigma \right\}$ of strings over the alphabet Σ_k_, all of length L′=2ℓ, that satisfy the following properties:

1. *For distinct characters* a,b∈Σ  *we have* $\mathrm{pre}(S_{a},\ell /3)\neq \mathrm{pre}(S_{b},\ell /3)$,
2. *for distinct characters* a,b∈Σ  *we have* $\mathrm{suf}(S_{a},\ell /3)\neq \mathrm{suf}(S_{b},\ell /3)$,
3. *for every pair of characters* a,b∈Σ  *(including the case when a = b) and every* r≥ℓ/3  *we have that* $\mathrm{pre}(S_{a},r)\neq \mathrm{suf}(S_{b},r)$.

Proof. Let us first observe that the total number of strings in ${\left(\Sigma_{k}\right)}^{\ell }$ is $\Theta (2^{{\;\text{log}\;}_{2}k100{\;\text{log}\;}_{2}|\Sigma |})=\Theta (|\Sigma {|}^{100{\;\text{log}\;}_{2}k})$ and therefore upper bounded by a polynomial in |Σ|. The algorithm initializes a list of strings L to contain all strings in ${\left(\Sigma_{k}\right)}^{\ell }$. The algorithm then removes from L all strings *T* such that there exists some r≥ℓ/3 such that pre(T,r)≠suf(T,r). For every r≥ℓ/3, the number of strings $T\in {\left(\Sigma_{k}\right)}^{\ell }$ such that the prefix of *T* of length *r* is equal to the suffix of *T* of length *r* is at most $k^{\frac{2\ell }{3}}$ (because fixing the $\frac{2\ell }{3}$ first characters uniquely determines the remaining $\frac{\ell }{3}$ characters). Thus, the total number of strings removed from the list in this initial cleaning step is at most $\ell \cdot k^{\frac{2\ell }{3}}$.

The algorithm then iterates over the characters a∈Σ one by one. When considering the character *a* the algorithm picks an arbitrary string still on the list L, calls it *S_a_*, and removes it from the list L. It then goes over all strings on the list L and removes all strings *T* such that $\mathrm{pre}(T,\ell /3)=\mathrm{pre}(S_{a},\ell /3)$, or $\mathrm{suf}(T,\ell /3)=\mathrm{suf}(S_{a},\ell /3)$, or there exists an r≥ℓ/3 such that $\mathrm{pre}(T,r)=\mathrm{suf}(S_{a},r)$ or $\mathrm{suf}(T,r)=\mathrm{pre}(S_{a},r)$.

There are at most $k^{\frac{2\ell }{3}}$ strings *T* such that $\mathrm{pre}(T,\ell /3)=\mathrm{pre}(S_{a},\ell /3)$ (since $\frac{\ell }{3}$ of the characters of *S* are uniquely determined by $\mathrm{pre}(S_{a},\ell /3)$). Similarly, there are at most $k^{\frac{2\ell }{3}}$ strings *T* such that $\mathrm{suf}(T,\ell /3)=\mathrm{suf}(S_{a},\ell /3)$, and at most $\ell \cdot k^{\frac{2\ell }{3}}$ strings *T* such that there exists an r≥ℓ/3 such that $\mathrm{pre}(T,r)=\mathrm{suf}(S_{a},r)$ or $\mathrm{suf}(T,r)=\mathrm{pre}(S_{a},r)$. Therefore, in each iteration (i.e. after selecting one string *S_a_*) the algorithm removes at most $4\ell \cdot k^{\frac{2\ell }{3}}$ strings from L

There are $|\Sigma |\leq 2^{\frac{\ell }{100}}$ iterations of the algorithm in total. Thus, in each iteration of the algorithm there are at least $k^{\ell }-4|\Sigma |\ell k^{\frac{2}{3}\ell }\geq \frac{k^{\ell }}{2}$ strings from L to choose the next string *S_a_* from. Thus, the algorithm successfully selects a string $S_{a}\in L$ for every character a∈Σ.

Finally, we can have the right idea argue that the set of strings $\left\{S_{a}\;:\;a\in \Sigma \right\}$ satisfy properties 1, 2 and 3. Suppose for distinct characters *a*, *b* we have that $\mathrm{pre}(S_{a},\ell /3)=\mathrm{pre}(S_{b},\ell /3)$ or $\mathrm{suf}(S_{a},\ell /3)=\mathrm{suf}(S_{b},\ell /3)$, or there exists an r≥ℓ/3 such that $\mathrm{pre}(S_{a},r)=\mathrm{suf}(S_{b},r)$ or $\mathrm{suf}(S_{a},r)=\mathrm{pre}(S_{b},r)$. Without loss of generality *S_a_* was selected before *S_b_*, and then *S_b_* would have been removed from L in the cleaning step immediately after *S_a_* was selected. This contradicts that *S_b_* was selected from the list L. Further, suppose that there exists an r≥ℓ/3 such that $\mathrm{pre}(S_{a},r)=\mathrm{suf}(S_{a},r)$ for some string *S_a_*. But then *S_a_* would have been removed in the initial cleaning step, again contradicting that *S_a_* was selected from L. This completes the proof of the claim.

The reduction algorithm now constructs the string S′ from *S* by replacing every character a∈Σ with the corresponding string *S_a_*. The reduction algorithm outputs the instance (Σ′,S′,L′). This concludes the construction. We claim that for every integer *t*, there exists a set T of *t* strings of length *L *=* *2 that cover *S* if and only if there exists a set T′ of *t* strings of length L′ that cover S′.

We begin by proving the forward direction of the above claim. Let T be a set of *t* strings of length *L* that cover *S*. We define $T\prime =\{S_{a}S_{b}\;:\;ab\in T\}$. Clearly |T′|=t. To see that T′ covers S′ consider an arbitrary position p′ in the string S′ and let $p=\left\lceil \frac{p\prime }{\ell }\right\rceil$. Since T covers *p* there is a string ab∈T that covers *p* in *S*. But then $S_{a}S_{b}\in T\prime$ covers all positions {ℓ·p−ℓ+1,ℓ·p−ℓ+2,ℓ·p} in S′. However p′∈{ℓ·p−ℓ+1,ℓ·p−ℓ+2,ℓ·p} and therefore T′ covers p′. Since p′ was an arbitrarily chosen position we conclude that T′ covers S′.

We now prove the reverse direction: if there exists a set T′ of *t* strings of length L′ that cover S′ then there exists a set T of *t* strings of length *L *=* *2 that cover *S*. Without loss of generality, every string T∈T′ is a substring of S′ of length 2ℓ (otherwise T′∖{T} also covers S′). Thus $T=\mathrm{suf}(S_{a},i)○S_{b}○\mathrm{pre}(S_{c},\ell -i)$ for some i∈{1,…,ℓ} and a,b,c∈Σ. Note that if i=ℓ then $T=S_{a}S_{b}$ for a,b∈Σ. Next we prove a claim about strings on this form.

Claim 3.4 *If* $\mathrm{suf}(S_{a},i)○S_{b}○\mathrm{pre}(S_{c},\ell -i)=\mathrm{suf}○(S_{a\prime },i\prime )○S_{b\prime }○\mathrm{pre}(S_{c\prime },\ell -i\prime )$  *then* i=i′  *and b = b. Furthermore, if* i≥ℓ/2  *then* a=a′*, otherwise* c=c′.

Proof Suppose that $\mathrm{suf}(S_{a},i)○S_{b}○\mathrm{pre}(S_{c},\ell -i)=\mathrm{suf}(S_{a\prime },i\prime )S_{b\prime }\;\mathrm{pre}(S_{c\prime },\ell -i\prime )$, and assume for contradiction that i≠i′. Without loss of generality we have i<i′.

We have that: $\mathrm{suf}(S_{a\prime },i\prime -i)=\mathrm{pre}(S_{b},i\prime -i),\;\mathrm{suf}(S_{b},\ell -i\prime +i)=\mathrm{pre}(S_{b\prime },\ell -i\prime +i)$, and $\mathrm{suf}(S_{b\prime },i\prime -i)=\mathrm{pre}(S_{c},i-i)$. Furthermore we have that $|\mathrm{suf}(S_{a\prime },i\prime -i)|+|\mathrm{suf}(S_{b},\ell -i\prime +i)|+|\mathrm{suf}(S_{b\prime },i\prime -i)|=\ell +i\prime -i\geq \ell$. Thus, at least one of $\mathrm{suf}(S_{a\prime },i\prime -i),\;\mathrm{suf}(S_{b},\ell -i\prime +i)$, or $\mathrm{suf}(S_{b\prime },i\prime -i)$ has length at least ℓ/3. However, either one of $\mathrm{suf}(S_{a\prime },i\prime -i),\;\mathrm{suf}(S_{b},\ell -i\prime +i)$, or $\mathrm{suf}(S_{b\prime },i\prime -i)$ having length at least ℓ/3 contradicts Property 3 of the set $\left\{S_{a}\;:\;a\in \Sigma \right\}$ of strings. We conclude that i=i′. But then $\mathrm{suf}(S_{a},i)=\mathrm{suf}(S_{a\prime },i),\;S_{b}=S_{b\prime }$ and $\mathrm{pre}(S_{c},\ell -i)=\mathrm{pre}(S_{c\prime },\ell -i)$. Property 1 implies that b=b′. If i≥ℓ/2 then Property 2 implies that a=a′. If i<ℓ/2 then ℓ−i≥ℓ/3 and therefore Property 1 implies that c=c′.

For every string T∈T′ we have that $T=\mathrm{suf}(S_{a},i)○S_{b}○\mathrm{pre}(S_{c},\ell -i)$ for some i∈{1,…,ℓ} and a,b,c∈Σ. By Claim 3.4 *i* and *b* are uniquely determined by *T*. If i≥n/2 then by Claim 3.4 *a* is also uniquely determined by *T*. In this case we add *ab* to the set T. If i<ℓ/2 then by Claim 3.4 *c* is also uniquely determined by *T*. In that case we add *bc* to the set T. In either case we add precisely one string of length 2 to T for each T∈T′. Thus |T|≤|T′| and it remains to prove that T covers *S*.

Consider an arbitrary position *p* of *S* and let abcde=S[p−2,p−1,p,p+1,p+2]. Let $p\prime =p\ell -\left\lfloor \frac{\ell }{2}\right\rfloor$, and let T∈T′ be such that T=S[x,x+1,…,x+L′−1] where p′∈{x,x+1,…,x+L′−1}. We have the following cases:

1.
$T=\mathrm{suf}(S_{a},i)○S_{b}○\mathrm{pre}(S_{c},\ell -i)$ , where ℓ−i≥ℓ/2, or
2.
$T=\mathrm{suf}(S_{b},i)○S_{c}○\mathrm{pre}(S_{d},\ell -i)$ , where i∈1,…,ℓ, or
3.
$T=\mathrm{suf}(S_{c},i)○S_{d}○\mathrm{pre}(S_{e},\ell -i)$ , where i≥ℓ/2.

In the first case bc∈T and S[p−1,p]=bc, in the second case bc∈T and S[p−1,p]=bc or cd∈T and S[p,p+1]=cd, while in the third case cd∈T and S[p,p+1]=cd. In either case the position *p* is covered by T, and so *S* is covered by T.

We remark that in the argument above, if p∈{1,2,|S|−1,|S|} then some of *a*, *b*, *d*, *e* are not properly defined. However this only restricts which of the cases 1, 2 and 3 may apply, one of them must still apply (for characters from *a*, *b*, *c*, *d*, *e* that are well defined). This concludes the proof.

#### 3.3 The Syotti algorithm

Our algorithm requires an index that can search for substrings in the input sequences $S_{1},\ldots ,S_{n}$ and report the locations of the exact matches. For this purpose, we use the combination of an FM-index (Ferragina and Manzini, 2005) and a generalized suffix array *GSA* (the suffix array of a set of strings (Shi, 1996)). Given a substring *x*, the FM-index is able to compute an interval [ℓ,r] such that the entries GSA[ℓ],…,GSA[r] give all starting positions of *x* in $S_{1},\ldots ,S_{n}$. That is, the entries of *GSA* in the interval are pairs (*t*, *p*) such that $S_{t}[p..p+|x|-1]=x$. We refer the interested reader to Mäkinen *et al.* (2015) for a detailed exposition of the techniques involved.

First, we construct the aforementioned data structures from the input sequences. The data structures are easily derived from the suffix array of the concatenation $S_{1}\$S_{2}\$\ldots \$S_{n}\#$, where $ is a separator character and # is an end sentinel. There exists there exists several linear-time construction algorithms (Puglisi *et al.*, 2007). In practice, we use the Divsufsort algorithm of Yuta Mori (https://github.com/y-256/libdivsufsort). Next, we initialize the set of bait sequences to an empty set, and initialize a bit vector for each sequence, of the same length as that sequence. The bit vector signifies which positions are covered by the bait sequences. Then, we perform a linear scan of each sequence, checking the bit vector at each position. When we come across a position that is not yet marked as covered, we add the substring of length L starting from that position into the set of bait sequences and update the bit vectors for all positions covered by the new bait. More specifically, we search for positions in all the sequences to which the new bait has Hamming distance less than or equal to θ. This search is done with a seed-and-extend heuristic based on the exact matching index as follows. For each seed k-mer x in the new bait sequence B, we find all occurrences of x in our input sequences. For each occurrence of x we consider the L-length sequence B′ that contains x in the same position as in B. If the Hamming distance of BandB′ is at most θ, we update the bit vector; otherwise, we move onto the next occurrence of x. After processing all seeds of B, we repeat the process for the reverse complement of B. Pseudocode is given in the Supplementary Material.

## 4 Experiments

We implemented Syotti in the C++ programming language. We use the Divsufsort and SDSL libraries (Gog *et al.*, 2014) for the construction of the FM-index and the generalized suffix array required by the Syotti algorithm. The implementation of Syotti is publicly available at https://github.com/jnalanko/syotti.

We compare the performance of CATCH, MrBait, and Syotti on three datasets: (i) MEGARES, which is a database containing 7868 AMR genes of total length 8 106 325 bp, publicly available at the MEGARes website: https://megares.meglab.org/download. (ii) VIRAL, which is a set of 422 568 viral genomes from CATCH (Metsky *et al.*, 2019), of total length 1 257 789 768 bp, publicly available at: https://github.com/broadinstitute/catch. (iii) BACTERIA, which is a custom (described in the Supplementary Material) set of 1000 bacterial genomes representing four foodborne pathogens containing 98 984 sequences of total length 3 040 260 476 bp.

As advised by the bait manufacturer Agilent, we set the bait length to 120 and tolerated 40 mismatches. The seed length in Syotti was set to 20. We ran CATCH, MrBait, and Syotti on these datasets on a server with two Intel Xeon Gold 5122 CPUs at 3.60 GHz for total of 8 physical cores and up to 16 threads with hyperthreading, 503 GB of available memory and a 3TB NVMe SSD for storage. The time is measured in wall-clock seconds. The experiments do not include BaitFisher because it requires a multiple sequence alignment of all the input sequences as input, but our sequences do not all align well to each other. Feeding multiple alignments computed with Clustal Omega (Sievers and Higgins, 2014) to BaitFisher produced extremely poor baits sets that had more nucleotides than the original sequences.

CATCH was run with the command line options -pl 120 -m 40 -l 120 -ps 60. CATCH also has a number of optional flags for tuning the behavior of the tool. We experimented with the options -cluster-and-design-separately 0.15 and -cluster-from-fragments 10 000, but did not see significant improvement on the results or the run time. We did not experiment with the option -filter-with-lsh-minhash, which might in retrospect have had significant impact on the running time. In any case, extensive tuning in the parameter space of CATCH is outside of the scope of this work.

MrBait was run with the options -A 120 -s tile = 0. MrBait also offers post-processing filtering using all-pairs global alignment from the external tool VSEARCH (Rognes *et al.*, 2016). This was feasible only for MEGARES because the time complexity of all-pairs alignment is quadratic. Syotti was run with options -L 120 -d 40 -r -t 16 -g 20.

We restricted all methods to using 72 h of time, and the available 503 GB of memory, and 3 TB of disk space. None of the methods exceeded the memory and disk limits before being terminated by the 72 h time limit. We analyzed the coverage of the produced bait sets by mapping the baits to the reference sequences using the seed-and-extend method with seed length 20, tolerating up to 40 mismatches. This is a similar method of analysis as the one used in the coverage analyzer of CATCH. We did not use the CATCH analyzer because it did not scale to our larger datasets. More specifically, it ran for more than 48 h on VIRAL, with memory usage increasing steadily up to 270 GB during the time. Based on the information printed by the program, we extrapolated that it might take more than 2 weeks to finish, and the memory usage would overflow the capacity of the machine before the end. To verify the comparability of our analyzer and the CATCH analyzer, we ran both analyzers on the first 10 000 baits of CATCH on MEGARes. Since the CATCH analyzer does not consider reverse complement matches, for the comparison we disabled reverse-complemented matching from our analyzer. Our analyzer reported 77.4% coverage, whereas the analyzer of CATCH reported 82.9%. The discrepancy is explained by how in our setup, the CATCH analyzer is likely regress to a randomized seed approach with a seed length of 10. Indeed, increasing the seed length to 10 increased our reported coverage to 84.6%, with the rest of the difference attributable to randomization. All analysis in this article was done consistently using our analyzer.

### 4.1 Results on antimicrobial resistance genes

**Fig. 1.**
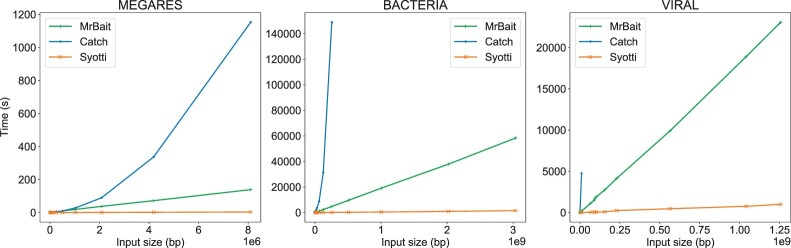
Time scaling on all three datasets

We ran all methods on exponentially increasing subsets of the MEGARes database that consists of 7868 AMR genes. The subsets were generated by shuffling the input sequences, and taking the first 1,2,4,8,… sequences. All methods were capable of being run on the complete dataset (i.e. 7868 AMR genes) within the given time and space limits. The memory, running time, and number of baits is given in Table 1. The scaling of the time usage is plotted in Figure 1.

**Table 1.** MEGARES

	Syotti			CATCH			MrBait		
Input length (base pairs)	Time (mm:ss)	Memory (MB)	Baits (count)	Time (mm:ss)	Memory (MB)	Baits (count)	Time (mm:ss)	Memory (MB)	Baits (count)
125 271	00:00	6	820	00:01	78	824	00:02	72	987
254 294	00:00	8	1372	00:04	104	1392	00:04	73	1981
508 459	00:00	11	2604	00:09	158	2635	00:09	76	3983
1 032 802	00:00	20	4633	00:28	260	4742	00:19	80	8093
2 090 517	00:00	35	7901	01:30	423	8121	00:37	88	16 374
4 187 569	00:01	67	13 099	05:37	735	13 489	01:12	101	32 764
8 106 325	00:03	125	20 976	19:13	1250	21 771	02:19	128	63 428

*Note*: Running time (Time), peak memory usage (Memory) and number of baits (Baits) for increasingly larger subsets of sequences from MEGARES (without VSEARCH filtering). The seconds are rounded down. Rows where all tools ran in <1 s have been removed. The full table is in the Supplementary Material.

Even though the worst case running time of Syotti is at least quadratic in the length of the input, the running time behaved approximately linearly. MrBait also behaved approximately linearly, but up to 40 times slower than Syotti. On the other hand, CATCH showed an upward-bending growth curve, which we speculate is due to having to update the profitability scores of candidate baits after every iteration, resulting in a quadratic time complexity in practice as well as in the worst case.

The size of bait sets of CATCH and Syotti had negligible difference, with the bait set of Syotti being consistently slightly smaller. MrBait produced two to three times larger bait sets than the other two. The memory usage of Syotti was the smallest of the three, however, the memory usage for all methods was <2 GB. The slope of the memory of MrBait was smaller than Syotti, which indicates that the memory of Syotti would grow past MrBait if the dataset size was still increased. We believe that MrBait’s good memory scaling is due to its use of a disk-based SQLite database.

**Fig. 2.**
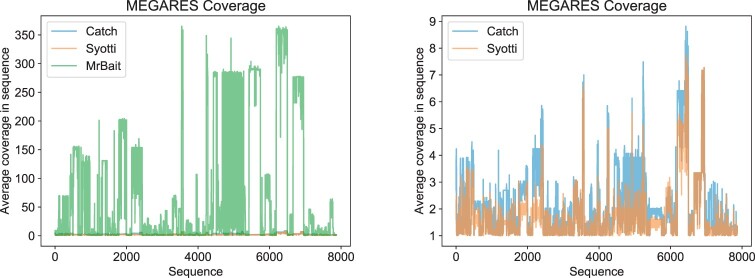
Average sequence coverage on the MEGARES dataset with all three methods (left) and with just CATCH and Syotti (right). The sequences are in the order of the MEGARes database. The coverage plot with all tools after filtering MrBait baits with VSEARCH is provided within the supplementary material.

Lastly, we evaluated the coverage of the baits. We note that the most optimal solution has a coverage value of 1, indicating sufficient likelihood of bait binding, but without redundancy (i.e. inefficiency) in the bait set. This is important for two reasons: first, multiple baits for the same position could create bait-bait interference during the binding process; and second, each bait is relatively costly, with a fixed upper limit on the total number of baits that can be manufactured. Coverage was analyzed by mapping the baits to the reference sequences with a seed-and-extend approach with a seed length of 20. The coverage of a position is defined as the number of mapped baits spanning that position with at most 40 mismatches. Figure 2 shows the mean coverage in each sequence of the database. Both Syotti and CATCH covered 100.0% of the nucleotide positions at least once, but MrBait covered only 96.4%. The bait set designed by MrBait was extremely inefficient, with many positions being covered by hundreds of baits (Fig. 2). The coverage efficiencies for CATCH and Syotti were comparable, with a slight advantage for Syotti (Fig. 2). Filtering the MrBait baits with VSEARCH and adding the command line parameters -f pw = 0.9,0.9 to MrBait, resulted in 22 230 baits with coverage 95.9%, but the run time increased drastically from 2 min 19 s to 4 h 22 min 36 s.

### 4.2 Results on bacterial strains and substrains

The aim of our second experiment was to study the scalability of the methods on pangenomes of clinically relevant bacterial species. Lacking a standard reference database of this type, we built our own, incorporating available sequences from foodborne pathogens *S.enterica*, *C.jejuni*, *E.coli* and *Enterococcus faecalis*. The purpose of the dataset is to cover as much of the known sequence diversity of these species as possible. The data were carefully selected and filtered to be suitable for the downstream application of foodborne pathogen detection. The full details of the process of compiling the dataset are available in the Supplementary Material. We note that this database and bait set from Syotti will be used in follow-up studies that aim to amplify these bacteria genomes in samples taken from food production facilities in order to test for foodborne pathogens.

To measure the scalability of the methods, we ran them on subsets of the data containing 1,2,4,8,16 … 65,536 sequences, as well as the full dataset consisting of all 98 984 sequences. The results are shown in Table 2. Syotti and MrBait were able to process all inputs, but CATCH hit the 72 hour time limit on inputs larger than 255 million base pairs.

**Table 2.** BACTERIA

	Syotti			CATCH			MrBait		
Input length (base pairs)	Time (hh:mm:ss)	Memory (MB)	Baits (count)	Time (hh:mm:ss)	Memory (MB)	Baits (count)	Time (hh:mm:ss)	Memory (MB)	Baits (count)
1 592 0441	00:00:08	237	72 035	00:29:32	4096	90 182	00:04:48	195	132 408
30 786 116	00:00:14	454	96 595	01:00:27	7575	134 112	00:10:00	316	256 041
62 502 135	00:00:27	917	123 541	02:26:56	14 234	181 825	00:18:44	572	519 838
125 063 199	00:00:52	1831	157 818	08:43:14	24 269	240 747	00:38:48	1076	1 040 174
254 576 853	00:01:45	3723	188 813	41:23:20	45 002	303 051	01:20:03	2071	2 117 436
505 422 833	00:03:31	7387	223 931	> 72 h	NA	NA	02:39:03	4056	4 203 752
1 003 934 029	00:07:11	14 673	267 890	NA	NA	NA	05:18:28	7980	8 349 888
2 018 459 352	00:15:50	29 496	324 797	NA	NA	NA	10:30:37	15 697	16 788 084
3 040 260 476	00:24:52	44 425	366 761	NA	NA	NA	16:13:43	23 615	25 286 576

*Note*: Running time (Time), peak memory usage (Memory) and number of baits (baits) for increasingly larger subsets of sequences from the BACTERIA dataset. ‘NA’ signifies that the dataset surpassed 72 h of running time. Rows where all tools took <30 min have been removed to save space. The full data table is in the Supplementary Material.

**Table 3.** VIRAL

	Syotti			CATCH			MrBait		
Input length (base pairs)	Time (hh:mm:ss)	Memory (MB)	Baits (count)	Time (hh:mm:ss)	Memory (MB)	Baits (count)	Time (hh:mm:ss)	Memory (MB)	Baits (count)
26 238	00:00:00	5	148	00:00:00	55	164	00:00:01	71	218
9 672 491	00:00:03	145	1103	01:19:09	2878	1065	00:02:43	130	67 054
66 970 374	00:00:37	1011	1640	> 72 h	NA	NA	00:18:59	577	522 683
911 29 015	00:00:55	1370	6016	NA	NA	NA	00:25:36	705	680 298
93 144 457	00:00:56	1400	6959	NA	NA	NA	00:28:41	734	696 193
103 290 818	00:01:00	1549	11 737	NA	NA	NA	00:32:01	799	773 385
155 140 833	00:01:28	2321	28 543	NA	NA	NA	00:45:40	1154	1 160 119
230 198 416	00:04:04	3424	60 539	NA	NA	NA	01:09:07	1549	1 557 350
564 924 375	00:07:45	8350	103 401	NA	NA	NA	02:45:21	3975	4 139 176
1 040 580 227	00:12:39	15 391	174 742	NA	NA	NA	05:14:59	7164	7 757 360
1 257 789 768	00:16:41	18 613	226 751	NA	NA	NA	06:24:44	8519	9 225 038

*Note*: Running time (Time), peak memory usage (Memory) and number of baits (count) for increasingly larger subsets of sequences from the VIRAL dataset. ‘NA’ signifies that the dataset surpassed 72 h of running time.

**Table 4.** Summary of the main results on the full datasets.

	MEGARES			BACTERIA			VIRAL		
	Syotti	CATCH	MrBait	Syotti	CATCH	MrBait	Syotti	CATCH	MrBait
Coverage	100%	100%	96.4%	100%	*	100%	100%	*	**
Number of baits	20 976	21 771	63 428	366 761	*	25 286 576	226 751	*	9 225 038
Time	3 s	19 min 13 s	2 min 19 s	24 min 52 s	> 72 h	16 h 13 min 43 s	16 min 41 s	> 72 h	6 h 24 min 44 s
Memory	125 MB	1250 MB	128 MB	44 425 MB	*	23 615 MB	18 613 MB	*	8519 MB

*Note*: VSEARCH filtering on MrBait on MEGARES brings the bait count down to 22 230 baits with coverage 95.9%, with a total run time of 4 h 22 min 36 s (the filtering was infeasible on the other two datasets due to the large number of baits from MrBait). (*) Unavailable due to CATCH not finishing in 72 h. (**) Unavailable due to the coverage analysis taking more than 72 h of running time due to the large number of baits and a large number of matches.

All three tools produced bait sets of similar size for the smaller prefixes. But as the length of the prefix increased, the bait sets produced by Syotti became significantly smaller than those produced by the other two tools. On the largest prefix that all tools were able to run, Syotti produced 189 thousand baits, CATCH 303 thousand and MrBait 2117 thousand. This showcases the ability of Syotti to deal with multiple bacterial pangenomes at once. On the full dataset, Syotti produced 367 thousand baits, whereas MrBait produced 25 million. Given the 220 000 limit provided by Agilent, two bait kits could be manufactured to include all our bait sequences, but over 113 bait kits would have to be manufactured for MrBait (which would cost more than 1 million USD for a single sample), making this bait set highly impractical.

The running times and memory usage of the tools showed similar trends to the first experiment on AMR sequences. Syotti is consistently the fastest by an order of magnitude. MrBait was eventually the most efficient in terms of peak memory, thanks to its SQLite database on disk, but it pays a large price for the disk-based approach in the form of a significantly slower running time.

**Fig. 3.**
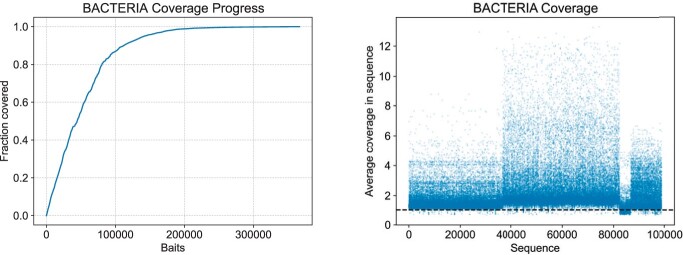
Left: Fraction of nucleotides covered by Syotti as the algorithm progresses on the full BACTERIA dataset. Right: average coverage of the reference sequences in BACTERIA after taking the first 200k baits produced by Syotti and filling gaps to maximum length 50. The dashed line shows coverage 1.0

We observe that very good trade-offs between coverage and number of baits are available by halting the greedy algorithm of Syotti before it reaches 100% coverage. Figure 3 shows the coverage as a function of the number of baits selected. For example, taking just the first 200k baits out of 367k already results in 98.8% coverage, whereas taking a *random* subset of 200k baits of the full set resulted in only 62.7% coverage. If long gaps in coverage are undesirable, we can add baits manually to patch the long gaps. For example, if we halt the greedy algorithm at 200k baits and then switch to filling the gaps such that the maximum gap length is 50 bp, we obtain a set of 275k baits with 99.6% coverage and with no gap longer than 50. Figure 3 shows the coverage plot for the 200k bait set after gap filling.

### 4.3 Results on viral strains and substrains

For our final experiment, we downloaded the set of human viral pangenomes published in the Github repository of CATCH in 2018. We studied scaling of the tools by shuffling the list of files and ran the tools on prefixes containing 1,2,4,8,16,…,512 files, as well as the whole dataset of 608 files. The results are shown in Table 3.

Syotti was again the fastest tool by an order of magnitude, producing 227k baits on the full dataset. MrBait again showed slow but approximately linear scaling, but the bait set was 40 times larger than the one produced by Syotti. Surprisingly, CATCH itself was unable to process prefixes longer than 1% of the full dataset within the time limit of 72 h. However, the Github page of CATCH contains pre-designed bait sets with 250k, 350k and 700k baits of length 75 for the dataset. The sets were designed by the authors of CATCH by running the tool separately for each species, and pooling the baits together with different parameters, optimizing the combined bait sets in the process.

To show CATCH in the best possible light, we compared the baits of Syotti against the published and optimized bait sets of CATCH. We used Syotti to generate baits of length 75, matching the bait length of the published data sets. The pooling method used with CATCH uses different values for the mismatch tolerance for different species to optimize the coverage. As Syotti does not support a varying mismatch tolerance, the tolerance of Syotti was set to 5, which is the maximum value in the parameters selected in Supplementary Table S1 of the manuscript of CATCH (Metsky *et al.*, 2019). With these settings, Syotti generated 684k baits.

**Fig. 4.**
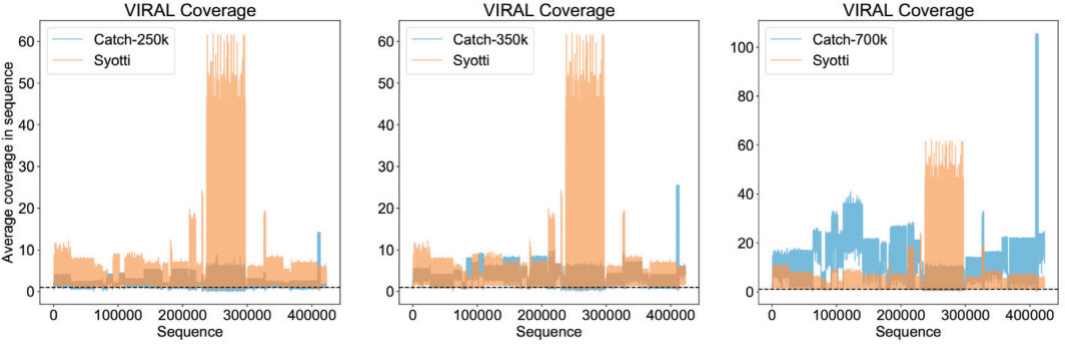
Coverage of the 684k baits generated by Syotti for the VIRAL dataset, versus and the published bait sets of sizes 250k, 350k and 700k from CATCH. The dashed line shows coverage 1.0

We analyzed the coverage of the bait sets allowing up to 5 mismatches. Coverage of the CATCH bait sets of size 250k, 350k and 700k were 84.1%, 90.6% and 97.5% respectively. The coverage of Syotti was 99.5%, with the remaining 0.5% being due to unknown N-characters in the data. The coverage plots of the bait set with 684k baits versus the CATCH bait sets are in Figure 4. Compared to the 700k baits of CATCH, Syotti had mostly lower coverage than CATCH (i.e. higher efficiency), except for one notable stretch of the input which finished with 60-fold coverage, where CATCH was able to keep the coverage to only 10-fold. This stretch represents the 59 686 rotavirus A strains in the database. Taking the first 250k baits generated by Syotti results in a coverage of 96.5%.

Table 4 provides a summary of all results described in Section 4.

## 5 Conclusion

In this article, we provide a formulation of designing baits and demonstrate that the problem is NP-hard even for deceptively simple instances, and provide a heuristic that works efficiently in practice. While both Syotti and CATCH use a kind of greedy heuristic, the heuristic of Syotti is designed to be much more efficiently implementable. However, we mention that CATCH was designed such that large datasets should be separated by strain or substrain into smaller datasets, have the baits designed on each smaller dataset, and then combined into a bait set for the complete dataset. This appears to work well if the input sequences are shorted (such as viral sequences) and the data can be separated into small subsets but does not work so well for whole bacterial genomes or datasets such that cannot be separated into small subsets by strain information. Our bacterial genomes is an example of this, as there are only four bacterial species that cannot easily be further separated. We conclude by suggesting some areas that warrant future work. First, a more sophisticated binding model than the Hamming distance could be plugged into the heuristic. Second, while our results rule out any reasonable fixed parameter tractable algorithms, there is potential that the problem admits an approximation algorithm. However, we conjecture that the problem remains hard even for a constant approximation. Third, other artifacts of the laboratory process are left for consideration, including considering the GC-content of the baits; designing baits in a manner that avoids contaminants; and designing baits to avoid bait-to-bait binding. Bait manufacturers currently perform some quality control measures related to GC-content, but bait design algorithms such as MrBait, CATCH and Syotti do not incorporate these considerations. Despite this, baits designed by Syotti and competing algorithms have been successfully used to capture and enrich viral and bacterial targets from real-world samples (Metsky *et al.*, 2019; Noyes *et al.*, 2017). Lastly, computationally designing baits in a manner that models and controls off-target binding is worth consideration.

## Supplementary Material

btac226_Supplementary_Data
