# Supplementary material for: Syotti: scalable bait design for DNA enrichment
Source: Bioinformatics. 2022 Jun 27;38(Suppl 1):i177–84. doi: 10.1093/bioinformatics/btac226 (PMC9235489; doi:10.1093/bioinformatics/btac226)
Supplement: btac226_Supplementary_Data [file btac226_supplementary_data.pdf]

1 Pseudocode for Syotti

Algorithm 1 The syotti algorithm.
Input: Sequences $S_1, \ldots, S_n$, bait length L, Hamming distance θ, seed length k
Output: Set of bait sequences covering $S_1, \ldots, S_n$

```
Construct the FM-index and the generalized suffix array GSA of S_1 ..., S_n
Baits ← ∅
for i = 1..n do
    for j = 1..|S_i| do
        if S_i[j] is not marked as covered then
            B ← S_i[j..j + L − 1]
            Baits ← Baits ∪{B}
            MarkMatches(B)
            MarkMatches(ReverseComplement(B))
return Baits.

function MarkMatches(B):
    for i = 1..|B| − k + 1 do
        x ← B[i..i + k − 1]
        [ℓ, r] ← FMindex.Search(x)
        % GSA-interval of the seed x
        for j = ℓ..r do
            % Locate the seed occurrence x = S_t[p..p + k − 1]
            t, p ← GSA[j]
            B′ ← S_t[p − i + 1..p − i + L]
            % Extend from the occurrence
            if d(B′, B) ≤ θ then
                Mark S_t[p − i + 1...p − i + L] as covered
```

There are some implementation details omitted from the pseudocode to avoid obfuscating the main idea of the algorithm. The string indices can go past the ends of the strings, but these corner cases are handled easily. That is, if a bait would run past the end of the string, we shift it back inside the string, and we ignore candidate matches from seeds that run past either end of the string. Also, if implemented directly as in the pseudocode, the seed-and-extend algorithm often ends up comparing the bait against the same position from multiple seeds in the same bait. To avoid this, we store the starting positions of the candidate matches in a set data structure that keeps only the distinct starting positions. We then run the Hamming distance computation on each distinct candidate match.

2 Details on how the Bacterial Dataset was Constructed

The pathogen set used was selected to represent whole genomes of public-health relevant food-borne isolates identified in food, animals, and human patients (clinical) using the NCBI Pathogen Detection tool [3], a repository developed for the curation of pathogenic bacterial genomic sequences. Salmonellae genomes were chosen to represent the serovars of greatest public health concern [1], including Enteriditis, Typhimurium, Infantis, Kentucky, Montevideo, and Dublin, representing an initial pool of 76,638 genomes and 3,486 SNP clusters. Shigatoxigenic Escherichia coli (STEC) isolates were chosen to include the O157:H7 serovars as well as the non-O157 STEC group (O26, O45, O103, O111, O121, and O145) including H-untypable, H-pending, H-undetermined, and non-motile types [2]. The resulting initial pool of STEC isolates represented 4,964 genomes and 1,164 SNP clusters. No specific subtype or serovar was chosen for Campylobacter jejuni (initial pool representing 60,227 genomes and 4,591 SNP clusters) or the Enterococci [4] (initial pool of 24,221 genomes and 1,774 SNP clusters of faecium and faecalis).

All isolates were then further filtered according to location (known U.S. and U.S. territories only), source (either from a clinical sample or from a domesticated animal or fowl collected pre- or post-slaughter, pre- or post-processing, and / or pre- or immediately post-fabrication stage in the food production system), collection date (2018–2020), sequence identity (i.e., availability of isolate-level WGS accession), and availability of SNP cluster assignment. Isolates were excluded if they originated from environmental samples (e.g., soil, water, sewage), ready-to-eat (RTE) products, or if any metadata variables were designated as ‘unknown’, ‘pending’, or left blank.

Stratified random sampling was conducted to select 1,000 total isolate genomes representative of the relative proportions of genera and serotypes in the final filtered pool of isolates (Salmonella enterica ssp. enterica: 322; STEC: 60; Campylobacter jejuni: 580; and Enterococci: 42). Corresponding WGS assemblies for all 1,000 isolates containing either a GCA or GCF prefix were retrieved from NCBI using the EDirect tool, and integrity of transferred data was ensured by evaluating file md5 checksum values.

3 Full Experimental Data Tables and Figures

All coverage plots, all time/mem plots. Full tables in case we delete some rows corresponding to the very smallest data prefixes.

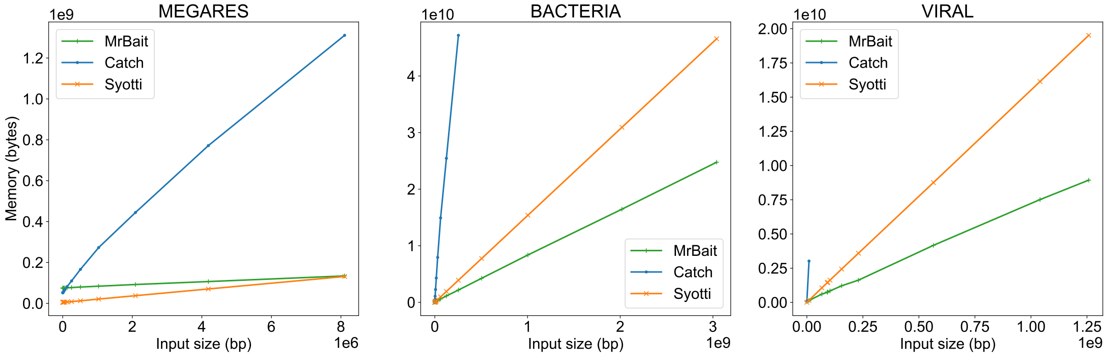

Figure 1: Memory scaling on all three datasets.

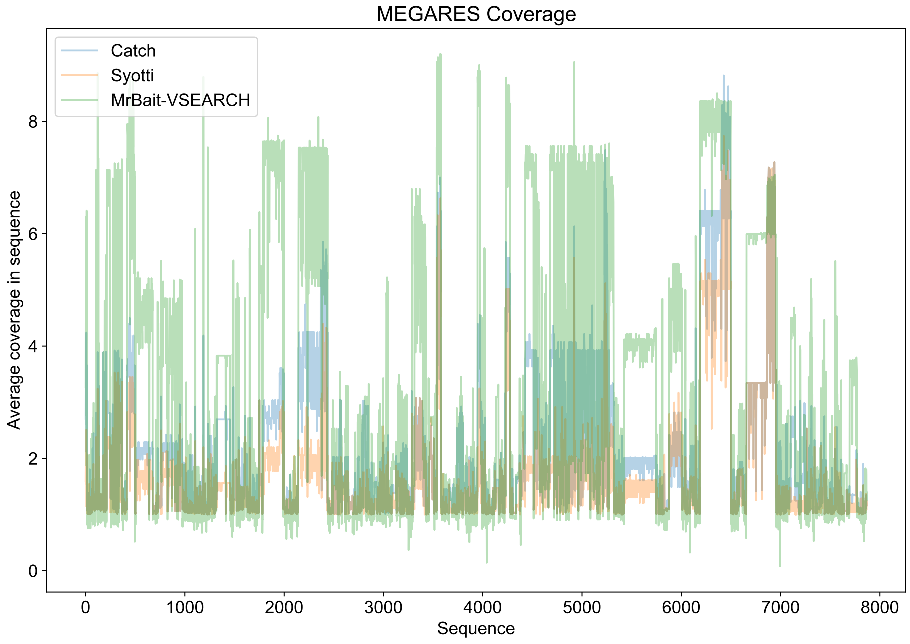

Figure 2: `MEGARES` coverage with VSEARCH filtering for MrBait.

	syotti			CATCH			MrBait		
Input length	Time	Memory (MB)	Baits	Time	Memory (MB)	Baits	Time	Memory (MB)	Baits
498	00:00	5	5	00:00	49	5	00:01	71	4
1320	00:00	5	12	00:00	49	12	00:00	71	10
2937	00:00	5	26	00:00	50	26	00:00	71	22
6672	00:00	5	59	00:00	50	59	00:00	71	52
14244	00:00	5	115	00:00	52	118	00:00	71	111
31758	00:00	5	249	00:00	56	253	00:01	71	249
60670	00:00	5	434	00:00	63	437	00:01	71	477
125271	00:00	6	820	00:01	78	824	00:02	72	987
254294	00:00	8	1372	00:04	104	1392	00:04	73	1981
508459	00:00	11	2604	00:09	158	2635	00:09	76	3983
1032802	00:00	20	4633	00:28	260	4742	00:19	80	8093
2090517	00:00	35	7901	01:30	423	8121	00:37	88	16374
4187569	00:01	67	13099	05:37	735	13489	01:12	101	32764
8106325	00:03	125	20976	19:13	1250	21771	02:19	128	63428

Table 1: Running time, memory usage and number of baits for increasing larger subsets of the `MEGARES` dataset. Times are in the format mm:ss.

	syotti			CATCH			MrBait		
Input length	Time	Memory (MB)	Baits	Time	Memory (MB)	Baits	Time	Memory (MB)	Baits
16794	00:00:00	5	140	00:00:00	52	140	00:00:01	71	139
27058	00:00:00	5	226	00:00:00	55	226	00:00:00	71	224
164612	00:00:00	7	1373	00:00:02	95	1373	00:00:03	72	1369
242069	00:00:00	7	2017	00:00:03	112	2017	00:00:05	73	2012
344651	00:00:00	9	2875	00:00:05	146	2875	00:00:06	74	2862
991610	00:00:00	19	8189	00:00:29	312	8264	00:00:19	80	8244
1572067	00:00:00	27	12821	00:01:01	485	12975	00:00:30	84	13067
3470929	00:00:02	55	25351	00:03:47	1022	27378	00:01:07	98	28857
8038466	00:00:04	122	48658	00:13:27	2158	56069	00:02:35	133	66861
15920441	00:00:08	237	72035	00:29:32	4096	90182	00:04:48	195	132408
30786116	00:00:14	454	96595	01:00:27	7575	134112	00:10:00	316	256041
62502135	00:00:27	917	123541	02:26:56	14234	181825	00:18:44	572	519838
125063199	00:00:52	1831	157818	08:43:14	24269	240747	00:38:48	1076	1040174
254576853	00:01:45	3723	188813	41:23:20	45,002	303051	01:20:03	2071	2117436
505422833	00:03:31	7387	223931	$>$ 72h	NA	NA	02:39:03	4056	4203752
1003934029	00:07:11	14673	267890	NA	NA	NA	05:18:28	7980	8349888
2018459352	00:15:50	29496	324797	NA	NA	NA	10:30:37	15697	16788084
3040260476	00:24:52	44425	366761	NA	NA	NA	16:13:43	23615	25286576

Table 2: Running time, memory usage and number of baits for increasing larger subsets of the `BACTERIA` dataset. The times are in the format hh:mm:ss.

References

[1] Food safety and inspection service (FSIS). Serotypes profile of Salmonella isolates from meat and poultry products January 1998 through December 2014. United States Department of Agriculture. 2015. https://www.fsis.usda.gov/sites/default/files/media$_{f}ile/2020 - 10/Salmonella - Serotype - Annual - 2014.pdf.Retrievedon28.November2021$.

[2] B. Devleesschauwer, S. M. Pires, I. Young, A. Gill, and S. E. Majowicz. Associating sporadic, foodborne illness caused by shiga toxin-producing escherichia coli with specific foods: a systematic review and meta-analysis of case-control studies. *Epidemiol. Infect.*, 147:e235, 2019.

[3] National Center for Biotechnology Information. NCBI pathogen detection project [Internet]. *Bethesda, MD. National Library of Medicine (US)*, 2016.

[4] R. Zaheer et al. Surveillance of Enterococcus spp. reveals distinct species and antimicrobial resistance diversity across a One-Health continuum. *Sci. Rep.*, 10(1):1–16, 2020.
